# Public health implications of lifestyle and sociocultural determinants of stroke risk and serum biochemical markers among older adults in northern Thailand

**DOI:** 10.3389/fneur.2026.1686339

**Published:** 2026-02-19

**Authors:** Pattaranai Chaiprom, Chatsuda Mata, Patana Nakatong, Benjawan Wongruen, Mayura Chaichumpoo, Pornanan Boonkorn

**Affiliations:** 1 Department of Community Public Health, Faculty of Science, Lampang Rajabhat University, Lampang, Thailand; 2 Faculty of Nursing, Lampang Rajabhat University, Lampang, Thailand; 3 Ban Nakot Subdistrict Health Promoting Hospital, Lampang Provincial Administrative Organization, Lampang, Thailand; 4 Ban Auan Subdistrict Health Promoting Hospital, Lampang Provincial Administrative Organization, Lampang, Thailand; 5 Department of Biology, Faculty of Science, Lampang Rajabhat University, Lampang, Thailand

**Keywords:** elderly health, lifestyle behavior, Northern Thailand, serum biomarkers, socio-cultural factors, stroke risk

## Abstract

**Introduction:**

Thailand is entering a fully aging society. The northern region has a unique socio-cultural landscape that may influence the risk of stroke. However, evidence on the interplay between lifestyle and socio-cultural factors and serum biomarkers in Thai elderly remains limited. The study aimed to investigate the correlation between socio-demographic, lifestyle, socio-cultural factors and stroke risk, as well as to evaluate the association between these factors and serum biochemical markers in elderly individuals residing in northern Thailand.

**Methods:**

A cross-sectional analysis was conducted among older adults (aged 60 years and older) with chronic conditions (*n* = 318). The Thai cardiovascular disease (CVD) risk assessment tool was used to stratify stroke risk into two groups. Data included demographics, education, occupation, health behaviors (diet: fatty, salty, sweet), clinical factors, and laboratory indicators (FBS, lipid profile, homocysteine). Univariate and multivariate logistic regression were used to assess stroke risk, and hierarchical linear regression (Models 1–3) was used to examine associations with serum biomarkers.

**Results:**

Of the 318 elderly participants, 229 (72%) were classified as at risk for stroke. Stroke risk was significantly associated with older age (70–79 years), hypertension, lower educational attainment, presence of underlying medical conditions, fasting glucose levels in the diabetic range, high total cholesterol, and elevated homocysteine levels. In the linear regression analysis, age was inversely associated with fasting glucose and cholesterol levels. Fat intake predicted higher cholesterol levels, sweet intake predicted higher fasting glucose levels, and salt intake predicted elevated homocysteine levels.

**Conclusion:**

These findings indicate that stroke risk among older adults in northern Thailand is influenced by biological aging and metabolic dysregulation. Educational disparities, possibly reflecting differences in health literacy, may further contribute to stroke vulnerability. Blood pressure, blood glucose, lipid parameters, and homocysteine levels highlight the role of interconnected metabolic pathways in shaping cerebrovascular risk.

## Introduction

The world is currently facing a growing aging population (1). Since 2021, Thailand has been officially recognized as an “aged society,” with more than 20% of the population aged 60 and over (2). By 2032, the elderly population in Thailand will exceed 28%, making Thailand a “super-aged society.” This demographic shift has affected the health behaviors, access to health services, and lifestyles of the elderly, especially in northern Thailand. This region is marked by cultural and social contexts that shape daily health behaviors. It is also a cause of several chronic diseases, including hypertension, diabetes, hyperlipidemia, arrhythmias, and chronic lifestyle conditions that can ultimately lead to cerebrovascular disease (3). This phenomenon has led to a significant global death toll, with the number of deaths increasing by 50% from 6.6 million to 9.7 million by 2050, particularly in low- and middle-income countries (4).

Ischemic stroke remains the most common type, accounting for approximately 50% of all cases, while hemorrhagic stroke accounts for approximately 20% and approximately 30% of cases are classified as indeterminate. A literature review found that predictors of stroke risk in the elderly include gender, with approximately 56% of stroke patients being male, with an average age of approximately 65 years, and underlying medical conditions such as hypertension (SBP ≥140/DBP ≥90 mm Hg), and reduced muscle mass and strength (5–9). Furthermore, central obesity and low HDL-C levels are also predictors of cardiovascular disease. Metabolic abnormalities, including hyperhomocysteinemia in cirrhosis due to metabolic fatty liver disease, are associated with low vitamin D and abnormal blood glucose and lipid levels, particularly in type II diabetes. A more precise risk indicator is needed (10–13).

Data from the World Stroke Organization found that every 1 min there are 30 new stroke patients, and on average one in four adults will have a stroke at least once in their life. For Thailand, data from the Medical and Health Data Warehouse System, Ministry of Public Health (HDC), in 2024 found a cumulative total of 363,688 stroke patients (14–16). The Public Health Statistics Report, Ministry of Public Health, Thailand, in 2023 found 37,947 deaths from stroke. Stroke is an emergency. Stroke remains the most common cause of death in Lampang Province, particularly in Mae Tha District. Elderly individuals are at high risk of developing chronic diseases, with incidence rates in 2021 of 48.54 per 100,000 hypertensive patients, 19.26 per 100,000 diabetic patients, and 119.42 per 100,000 first-time stroke patients (17). These statistics demonstrate the importance of culturally relevant behavioral strategies. Prevention should focus on risk factors to mitigate the increasing incidence of stroke.

Despite extensive research on stroke risk factors, some remain understudied. This scenario is particularly true for cystathionine levels, an amino acid formed between methionine and cysteine, which has been shown to significantly correlate with high Hcy levels and stroke (18, 19). Furthermore, risk factors related to community cultural contexts may play a significant role in health behaviors and disease development. Therefore, identifying these additional risk factors is essential to comprehensively elucidate disease mechanisms, enhance screening and prevention programs tailored to the local context, and develop targeted health care measures that better respond to community differences. This study aimed to assess stroke risk among elderly individuals in northern Thailand and to further assess whether social and behavioral factors, particularly health behaviors and social practices, are related to other serum biochemical markers of stroke risk.

## Methods

### Study design

The objective was to study the risk factors for stroke, including social behavioral determinants and serum biochemical indexes in elderly patients with chronic diseases, in terms of lifestyle factors and traditional Thai health care activities in Northern Thailand.

### Sample size

The sample size calculation was performed using Taro Yamane's (1967) formula (20), which yielded a total of 318 participants. In addition, adequacy for multivariable logistic regression was evaluated using the commonly recommended rule of at least 10 outcome events per predictor variable (EPV). With 229 participants classified as at risk for stroke, the number of events was sufficient to support the predictors included in the adjusted model. The inclusion criteria were 1) elderly men and women aged 60 years and above, 2) diagnosed with any chronic disease, including hypertension and/or diabetes, 3) no history of cerebrovascular disease, 4) able to read and speak Thai, and 5) willing to participate. Stratified random sampling was used to maintain representativeness from each village, recognizing the different social structures and community organizations existing in Northern Thailand. After stratification by population proportion, simple random sampling was used to select a random list of eligible elderly persons from each village.

### Research instruments

The questionnaire consisted of five sections: Part 1 was sociodemographic and clinical information (14 items), including age, anthropometric measurements, marital status, education level, occupation, monthly income, underlying diseases, current alcohol consumption (including local Thai alcoholic beverages), family history of stroke, and exercise (including our traditional exercise). Part 2 was cardiovascular disease risk assessment comprised of eight domains evaluating clinical risk factors: age, gender, blood pressure, waist circumference, height, diabetes history, smoking status, and overall cardiovascular disease risk. Smoking assessment included conventional tobacco products commonly used in Northern Thailand. Cardiovascular risk stratification was conducted using the validated Thai CVD risk assessment tool (21). Stroke risk was determined using the validated Thai CVD risk assessment tool. In accordance with Thai Ministry of Public Health guidelines, participants with a calculated 10-year cardiovascular disease (CVD) risk of ≥10% were classified as at risk of stroke, whereas those with a risk of < 10% were classified as not at risk of stroke. This cutoff was used to dichotomize the outcome variable for subsequent logistic regression analyses. Part 3 was a stroke knowledge assessment, including their causes, symptoms, prevention, and how they are portrayed by the cultural beliefs in Northern Thai communities, which was evaluated by 10 items. The response alternatives were correct (one point) or incorrect (0 points). Knowledge level was classified as high (≥ 8 points), moderate (6–7 points), or low (< 6 points). Part 4 was perceptions of stroke prevention. 10 items scaled participants' attitudes toward stroke prevention, including cultural perspectives on preventative healthcare, on a 5-point Likert scale (from strongly agree (5) to strongly disagree) (1). Scoring for negative statements was reversed. Part 5 was behavior assessment. Fourteen items evaluated health-related behaviors across three domains: dietary practices, healthcare-seeking behavior, and physical activity engagement. All items were scored using a 5-point Likert scale. Questionnaires for Parts 3–5 were developed based on the Biopsychosocial model and literature review.

The laboratory evaluation assessed seven clinical biomarkers critical for cardiovascular and metabolic risk assessment: low-density lipoprotein cholesterol (LDL), high-density lipoprotein cholesterol (HDL), triglycerides, total cholesterol, fasting blood glucose and homocysteine levels. These parameters provided comprehensive insights into participants' lipid profiles, glycemic control, and additional cardiovascular risk factors. All analyses were performed by certified medical technologists following standardized operating procedures within accredited laboratory facilities.

### Cultural adaptation and instruments validation

Consensus content validity was established through expert review, whereby all instruments were submitted to three specialists in geriatric healthcare and Thai cultural health practices for evaluation of cultural appropriateness, content comprehensiveness, format clarity, and contextual suitability. Following expert feedback incorporation, the instruments underwent iterative revisions before pilot testing with 30 non-sample participants from comparable communities to ensure comprehension and cultural relevance. The reliability assessment indicated robust psychometric features. The knowledge exam yielded a KR20 coefficient of 0.85, while the attitudes and behavioral practice tests produced Cronbach's alpha values of 0.91 and 0.95, respectively. The final tools were adapted to accommodate the distinctive cultural characteristics of Northern Thailand, including dietary habits, preferred forms of exercise, and the health beliefs and practices of its inhabitants.

## Ethical considerations

This study was approved by the Human Research Ethics Committee of Boromarajonani College of Nursing, Nakhon Lampang (E2566-133) on January 16, 2024. Data collection was preceded first by verbal and then written informed consent, with highlights of local approaches for each consent process.

## Statistical analysis

Statistical analyses were performed using IBM SPSS Statistics version 27.0. Descriptive statistics were applied to summaries the participants' sociodemographic characteristics, behavioral factors, and biological markers. Categorical variables were presented as frequencies and percentages, whereas continuous variables were reported as means with standard deviations (mean ± SD). To identify factors independently associated with stroke, both univariate and multivariate logistic regression analyses were conducted, with results expressed as adjusted odds ratios (AOR) and 95% confidence intervals (CI). Variables that showed a *p-value* less than 0.05 in the univariate analysis were subsequently included in the multivariate logistic regression model to control for potential confounding effects. In addition, hierarchical multiple linear regression analyses were performed to examine the relationships between sociodemographic characteristics, health behaviors, biological factors, and serum biochemical marker. The regression analysis was constructed sequentially in three models. Model 1 included demographic variables (age and gender). Model 2 incorporated socioeconomic factors, including occupation, and Model 3 included dietary behavioral elements, particularly the intake of sugary, fatty, and salty meals. The stepwise approach allowed us to evaluate how the addition of socioeconomic variables and lifestyle-related factors influenced the magnitude and significance of associations between predictors and biomarker outcomes. Regression coefficients (B), 95% confidence intervals (CI), and *p*-values were reported for each model. Statistical significance was set at *p-value* < 0.05.

## Result

Table 1 presents the distribution of stroke risk among the 318 elderly participants, of whom 229 (72%) were classified as at risk and 89 (28%) as not at risk. The table further reports the results of univariate and multivariate logistic regression analyses evaluating sociodemographic, lifestyle, clinical, and laboratory factors associated with stroke risk.

**Table 1:** Logistic regression analysis of sociodemographic, clinical, lifestyle, and laboratory factors associated with stroke risk among study participants (*n* = 318).

**Variable factors**	**Total (*n* = 318)**	**Risk of stoke**		**Crude**		**Adjusted**	
		**No (*****n*** = **89)**	**Yes (*****n*** = **229)**				
		***n*** **(%)**		**COR (95% CI)**	***p*** **-value**	**AOR (95% CI)**	***p*** **-value**
**Personal information**							
**Gender**							
Female	211 (66.4)	65 (30.8)	146 (69.2)	Ref.			
Male	107 (33.6)	24 (22.4)	83 (77.6)	1.54 (0.89–2.64)	0.12		
**Age (years)**							
60–69	195 (61.3)	78 (40.0)	117 (60.0)	Ref.			
70–79	94 (29.6)	7 (7.5)	87 (92.5)	8.29 (3.64–18.84)	< 0.01	6.31 (2.56–15.55)	< 0.01
≥80	29 (9.1)	4 (13.8)	25 (86.2)	4.17 (1.40–12.44)	0.01	3.27 (0.91–11.81)	0.07
**Blood pressure**							
Normal (120–139/80–89 mmHg)	160 (50.3)	62 (38.8)	98 (61.2)	Ref.			
High (140–159/90–99 mmHg)	106 (33.3)	21 (19.8)	85 (80.2)	2.56 (1.44–4.55)	< 0.01	2.17 (1.12–4.20)	0.01
Very High (160–179/100–109 mmHg)	52 (16.4)	6 (11.5)	46 (88.5)	4.85 (1.96–12.03)	< 0.01	3.53 (1.30–9.57)	0.01
**BMI**							
Normal (18.5–22.9)	145 (45.6)	44 (30.3)	101 (69.7)	Ref.			
Overweight (> = 23)	173 (54.4)	45 (26.0)	128 (74.0)	1.24 (0.76–2.02)	0.39		
**Marital status**							
Single	32 (10.1)	9 (28.1)	23 (71.9)	Ref.			
Married	202 (63.5)	60 (29.7)	142 (70.3)	0.93 (0.41–2.12)	0.86		
Separated	84 (26.4)	20 (23.8)	64 (76.2)	1.25 (0.49–3.14)	0.63		
**Education level**							
Primary elementary school	48 (15.1)	30 (62.5)	18 (37.5)	Ref.			
Uneducated	270 (84.9)	59 (21.9)	211 (78.1)	5.96 (3.11–11.44)	< 0.01	3.27 (1.52–7.00)	< 0.01
**Career**							
Employed	223 (70.2)	76 (34.1)	147 (65.9)	Ref.			
Unemployed	95 (29.8)	13 (13.7)	82 (86.3)	3.26 (1.71–6.23)	< 0.01	1.62 (0.75–3.52)	0.22
**Congenital disease**							
No	47 (14.8)	26 (55.3)	21 (44.7)	Ref.			
Yes	271 (85.2)	63 (23.3)	208 (76.7)	4.09 (2.16–7.76)	< 0.01	3.41 (1.54–7.54)	< 0.01
**Family history of stroke**							
No	280 (88.1)	82 (29.3)	198 (70.7)	Ref.			
Yes	38 (11.9)	7 (18.4)	31 (81.6)	1.83 (0.78–4.33)	0.17		
**Exercise**							
Yes	187 (58.8)	58 (31.0)	129 (69.0)	Ref.			
No	131 (41.2)	31 (23.6)	100 (76.4)	1.45 (0.87–2.41)	0.15		
**Smoking**							
No	269 (84.6)	75 (27.9)	194 (72.1)	Ref.			
Yes	49 (15.4)	14 (30.4)	35 (69.6)	1.00 (0.40–2.54)	0.10		
**Alcohol consumption**							
No	241 (75.8)	58 (24.1)	183 (75.9)	Ref.			
Yes	77 (24.2)	31 (40.3)	46 (59.7)	2.13 (1.24–3.66)	0.01	1.05 (0.52–2.10)	0.90
**Medical checkup**							
Regularly	283 (89.0)	76 (26.9)	207 (73.1)	Ref.			
Occasionally	35 (11.0)	13 (37.2)	22 (62.8)	0.62 (0.30–1.30)	0.20		
**Medication consumption**							
Complete	289 (90.9)	76 (26.3)	213 (73.7)	Ref.			
Incomplete	29 (9.1)	13 (44.8)	16 (55.2)	0.44 (0.20–0.96)	0.04	2.08 (0.71–6.06)	0.18
**Knowledge, attitudes, practice level information on stroke**							
**Knowledge level**							
High	272 (85.5)	75 (27.6)	197 (72.4)	Ref.			
Low	46 (14.5)	14 (30.4)	32 (69.6)	0.6 (0.58–2.27)	0.69		
**Attitude level**							
Negative attitudes	181 (56.9)	48 (26.5)	133 (73.5)	Ref.			
Positive attitudes	137 (43.1)	41 (29.9)	96 (70.1)	1.18 (0.72–1.94)	0.50		
**Dietary behavior**							
Healthy eating behavior	154 (48.4)	53 (34.4)	101 (65.6)	Ref.			
Unhealthy eating behavior	164 (51.6)	36 (22.0)	128 (78.0)	1.87 (1.14–3.07)	0.01	1.42 (0.78–2.58)	0.25
**Laboratory blood test**							
**HbA1C level**							
Mean ± SD	7.02 ± 1.85						
Range	4.50–15.90					
Normal range (4.2–6.3%)	124	38 (30.7)	86 (69.3)	Ref.	0.44		
Diabetes	194	51 (26.3)	143 (73.7)	1.32 (0.65–2.68)			
**FBS level**							
Mean ± SD	146.10 ± 49.90						
Range	88–369					
Normal range (70–105 mg/dL)	145	37 (25.5)	108 (74.5)	Ref.			
Diabetes	173	52 (30.1)	121 (69.9)	2.05 (0.99–4.21)	0.05	3.00 (1.35–6.66)	< 0.01
**Cholesterol level**							
Mean ± SD	180.90 ± 43.13						
Range	94–335					
Optimal (< 200 mg/dL)	89	46 (51.7)	43 (48.3)	Ref.			
High	229	43 (18.8)	186 (81.2)	3.62 (1.82–7.19)	< 0.01	3.07 (1.41–6.68)	< 0.01
**Triglyceride level**							
Mean ± SD	147.47 ± 70.97						
Range	52–377					
Optimal (< 150 mg/dL)	212	58 (27.4)	154 (72.6)	Ref.			
High	106	31 (29.3)	75 (70.7)	3.07 (1.25–7.59)	0.02	2.21 (0.84–5.80)	0.12
**LDL level**							
Mean ± SD	113.64 ± 38.44						
Range	36–248					
Optimal (< 100 mg/dL)	236	57 (24.2)	179 (75.8)	Ref.			
High	82	32 (39.0)	50 (61.0)	1.68 (0.71–3.97)	0.24		
**HDL**							
Mean ± SD	52.36 ± 11.57						
Range	32–90					
Optimal (< 40 mg/dL)	207	58 (28.0)	149 (72.0)	Ref.			
High	111	31 (27.9)	80 (72.1)	3.32 (1.35–8.19)	0.01	2.69 (0.94–7.70)	0.07
**Homocysteine Hormone**							
Mean ± SD	13.8 ± 5.7						
Range	5.65–30.51					
Optimal (5.08–15.39 umol/L)	209	57 (27.3)	152 (72.7)	Ref.			
High	109	32 (29.4)	77 (70.6)	2.63 (1.12–6.18)	0.03	3.77 (1.51–9.43)	< 0.01

### Sociodemographic factors

Age was strongly associated with stroke risk. Compared with older adults aged 60–69 years, those aged 70–79 years had significantly higher odds of being at risk (AOR = 6.31, 95% CI: 2.56–15.55, *p* < 0.01). Participants aged ≥80 years also demonstrated increased risk, although the association did not reach statistical significance in the adjusted model (AOR = 3.27, 95% CI: 0.91–11.81, *p* = 0.07). Education level was another significant predictor. Older adults with no formal education were more likely to be at risk for stroke than those with primary education (AOR = 3.27, 95% CI: 1.52–7.00, *p* < 0.01). The presence of congenital or underlying disease was also a significant predictor of stroke risk, as individuals with such conditions had markedly higher odds of being classified as at risk compared with those without (AOR = 3.41, 95% CI: 1.54–7.54, *p* < 0.01).

### Clinical characteristics

Blood pressure showed a clear graded relationship with stroke risk. Individuals with high blood pressure (AOR = 2.17, 95% CI: 1.12–4.20, *p* = 0.01) and very high blood pressure (AOR = 3.53, 95% CI: 1.30–9.57, *p* = 0.01) were significantly more likely to be classified in the stroke-risk group compared with those with normal blood pressure. The presence of underlying chronic conditions also increased risk. Participants with congenital or chronic diseases were more than three times as likely to be in the at-risk group (AOR = 3.41, 95% CI: 1.54–7.54, *p* < 0.01).

### Lifestyle factors

Unhealthy eating behavior was associated with stroke risk in the crude model (COR = 1.87, *p* = 0.01), but this association was not statistically significant after adjustment (AOR = 1.42, *p* = 0.25). Neither physical activity, smoking, nor alcohol consumption showed significant adjusted associations.

### Laboratory indicators

Metabolic biomarkers demonstrated strong associations with stroke risk. Participants with fasting blood glucose in the diabetic range had significantly higher odds of stroke risk (AOR = 3.00, 95% CI: 1.35–6.66, *p* < 0.01). Similarly, those with high total cholesterol levels were at increased risk (AOR = 3.07, 95% CI: 1.41–6.68, *p* < 0.01). Elevated homocysteine levels were also associated with increased stroke risk (AOR = 3.77, 95% CI: 1.51–9.43, *p* < 0.01), supporting the role of vascular biochemical markers in risk stratification.

### Associations between sociodemographic and lifestyle factors and biomarkers

Multiple linear regression models examining predictors of fasting blood sugar (FBS), cholesterol, and homocysteine levels are shown in Table 2.

**Table 2:** Hierarchical linear regression models of demographic, socioeconomic, and dietary factors on biochemical markers (FBS, Cholesterol, Homocysteine).

**Sociodemographic**	**FBS**			**Cholesterol**			**Homocysteine**		
	**B**	***p-value***	**95%CI**	**B**	***p-value***	**95%CI**	**B**	***p-value***	**95%CI**
**Model 1**									
Age	−1.66	< 0.01	−2.67; −0.67	−1.38	< 0.01	−2.30; −0.46	0.09	0.14	−0.03; 0.21
Gender	−32.83	< 0.01	−43.96; −21.70	−15.33	< 0.01	−25.34; −5.33	3.43	< 0.01	2.13; 4.73
**Model 2**									
Age	−1.47	< 0.01	−2.49; −0.44	−1.25	< 0.01	−2.15; −0.35	0.10	0.12	−0.03; 0.22
Gender	−34.28	< 0.01	−45.45; −23.11	−18.59	< 0.01	−28.43; −8.73	3.65	< 0.01	2.33; 4.98
Occupational	−17.68	< 0.01	−28.29; −7.07	−18.77	< 0.01	−28.13; −9.42	0.21	0.75	−1.05; 1.47
**Model 3**									
Age	−1.81	< 0.01	−2.81; −0.80	−1.54	< 0.01	−2.43; −0.64	0.11	0.06	−0.01; 0.23
Gender	−28.25	< 0.01	−39.63; −16.87	−22.04	< 0.01	−32.16; −11.92	3.41	< 0.01	2.07; 4.75
Consumption of fatty foods	−16.10	0.03	−30.27; −1.93	31.06	< 0.01	18.46; 43.65	0.25	0.77	−1.42; 1.91
Consumption of salty foods	7.19	0.26	−5.42; 19.80	−1.20	0.83	−12.41; 10.02	1.94	0.01	0.46; 3.43
Consumption of sweet foods	23.52	< 0.01	11.48; 35.56	−9.04	0.09	−19.74; 1.69	−1.05	0.15	−2.46; 0.37

### Fasting blood sugar

Across all three models, age was inversely associated with FBS (Model 3: B = −1.81, *p* < 0.01). Gender showed a significant association, with males having lower FBS levels than females (B = −28.25, *p* < 0.01). In Model 3, sweet food consumption was positively associated with FBS (B = 23.52, *p* < 0.01), indicating that higher intake of sweets predicts elevated fasting glucose.

### Cholesterol

Age was negatively associated with cholesterol (Model 3: B = −1.54, *p* < 0.01). Male gender predicted lower cholesterol levels (B = −22.04, *p* < 0.01). Dietary intake showed meaningful effects: fat consumption significantly increased cholesterol (B = 31.06, *p* < 0.01), consistent with metabolic risk pathways.

### Homocysteine

Gender remained a strong predictor of homocysteine, with males showing higher levels (B = 3.41, *p* < 0.01). Among dietary variables, salt consumption was significantly associated with elevated homocysteine levels (B = 1.94, *p* = 0.01). No significant associations were observed for age, fatty food intake, or sweet food intake in the final model.

## Discussion

This study provides an in-depth examination of the association between socio-demographic factors, specific lifestyles of elderly people in northern Thailand, and biochemical factors affecting stroke risk. The findings are consistent with global evidence regarding vascular degeneration, specifically decreased arterial stiffness and lower metabolic status. Furthermore, some individuals exhibit premature vascular degeneration associated with endothelial dysfunction, hypertension, hypoglycemia, and dyslipidemia, leading to an increased risk of stroke (22, 23). Although this research indicates a positive association between age and elevated homocysteine levels, adjusted models found that age was not a significant predictor of homocysteine levels, suggesting that other biological or behavioral factors may better explain homocysteine variability in this elderly population. Furthermore, a clear gender difference was observed, with men having significantly higher homocysteine levels than women, consistent with research from northeast China showing that elevated homocysteine levels are more prevalent in males than in females (24). Higher homocysteine levels negatively impact cardiovascular outcomes, with men having a higher risk than women (25). These patterns indicate the importance of targeted biochemical screening in older men, who may have increased vascular fragility.

Education level is an important social factor in stroke risk. Participants without formal education are significantly more likely to be classified as high-risk. Such evidence has led to research focusing on promoting health knowledge among this population to increase understanding of basic health care, including medication use and health care for those with underlying conditions, such as controlling hypertension (26). These findings highlight the importance of social factors, especially health knowledge, in determining cardiovascular disease outcomes. Studies have also shown that sugary food consumption significantly predicts fasting blood glucose levels, while salt consumption predicts higher homocysteine concentrations. Both patterns reflect culturally ingrained dietary habits that impact metabolism. Specifically, dietary habits and culture in northern Thailand play a significant role in metabolic health. Sticky rice, a staple food in the region, has a high glycemic index and glycemic load, resulting in rapid spikes in blood glucose levels and increased insulin requirements. Several studies have documented sticky rice consumption as associated with impaired glycemic control and increased risk of metabolic disorders in both rural and urban Thai populations (27, 28). Furthermore, dietary changes, including consumption of processed foods, sugary snacks, and high-sodium fermented foods, are also contributing factors to higher rates of obesity, diabetes, and metabolic syndrome (29).

In this study, high blood pressure is still the most important sign of stroke risk. People with high blood pressure are higher risk, which is in line with the fact that high blood pressure is the most important risk factor for stroke that can be controlled (30, 31). Thai eating habits have a direct effect on blood pressure. Foods that are fermented, instant noodles, and spices are all high in sodium, which can raise blood pressure (32). Research on elderly individuals in Thailand shows that sodium intake is a key factor in hypertension, which points to the importance of culturally specific risk factors in the region (33). Along with blood pressure, metabolic biomarkers were also strongly linked to the risk of stroke. People who had high total cholesterol levels and people who had high fasting blood glucose levels were much more likely to have a stroke. These results are in line with what we know about how high blood sugar, high cholesterol, oxidative stress, and endothelial dysfunction can all raise the risk (34, 35). High levels of homocysteine can predict stroke risk on their own and can also cause damage to blood vessels by damaging the endothelium, causing blood clots, and triggering inflammation. In general, these results show how the metabolic processes that make the elderly more likely to have cerebrovascular fragility are all linked to each other (36). In addition to the significant predictors identified, several factors traditionally associated with stroke risk did not show significant associations in this study. According to the logistic regression model, gender, BMI, marital status, employment status, family history of stroke, smoking, alcohol consumption, physical activity, and overall dietary behavior were not independent predictors of stroke risk after adjustment. These null findings indicate that after controlling for significant metabolic and clinical predictors such as blood pressure, fasting glucose, cholesterol, and homocysteine, the impact of demographic or lifestyle factors diminishes in this elderly Thai population. Likewise, several common lipid markers, including LDL, HDL, and triglycerides, did not significantly predict stroke risk, despite the significance of total cholesterol. Findings from the linear regression models (Table 2) further clarify which factors did not meaningfully contribute to biochemical variation. Age, fatty food intake, and sweet food intake were not significant predictors of homocysteine levels, and salty or sweet food intake did not predict cholesterol concentrations. Occupational status also did not significantly predict homocysteine. These patterns indicate that behavioral factors do not always influence all biomarkers, and their effects may be specific. However, when considered together, behavioral factors alone cannot fully explain the variability in cardiovascular risk. Metabolic dysfunction and clinical status exert a more significant impact on stroke risk in older adults in northern Thailand. Interpreting these results through a biosocial-psychological framework (37) highlights the dynamic interaction between biological mechanisms (age, hypertension, hyperglycemia, dyslipidemia, and homocysteine), behavioral factors (dietary patterns), and social factors (education level, household structure, and socioeconomic changes). Furthermore, lifestyle behaviors among older adults influence not only physical health but also psychological well being. Evidence from Hamidi et al. (38) demonstrated that psychological well being mediates the relationship between a health-promoting lifestyle and reduced death anxiety among seniors with COVID-19 experience. This finding supports the broader notion that lifestyle modification can produce benefits across both physiological and psychosocial domains, reinforcing the need for integrated health education and behavioral interventions for older adults (38). The convergence of these factors demonstrates that health outcomes in this population are determined by complex and interdependent influences, reflecting the broader social and cultural context of aging in northern Thailand.

This study has several limitations. Firstly, the cross-sectional study design limits the ability to conclude causal relationships between socio-demographic, behavioral, and biological factors and stroke risk or biochemical outcomes. Therefore, longitudinal or intervention studies are needed to confirm these relationship pathways. Secondly, the requirement for participants to be literate in Thai may result in the exclusion of ethnic minorities or illiterate groups, affecting the ability to generalize to the entire population. Thirdly, self-reported behavioral variables may be subject to recognition bias or social pressure, which may reduce data accuracy. In addition, the inclusion of a large number of independent variables may reduce the explanatory power of the model. Future research should use larger and more diverse sample sizes to increase the reliability of the results. However, despite these limitations, this study suggests that important risk factors, including blood pressure, fasting blood glucose levels, cholesterol, and homocysteine, can be detected through primary care screening services. Disease prevention strategies in northern Thailand should integrate biochemical screening with contextually appropriate health education programs, including nutritional counseling, proactive screening in temple activities, mobile medical units, and village health volunteer networks, which may be an effective approach. Furthermore, we should prioritize elderly individuals with low levels of education, as they represent a particularly high-risk group. Future research should focus on long-term studies or experimental interventions that take into account cultural contexts to elucidate causal mechanisms and evaluate appropriate prevention strategies in the area. The need for integrated and forward-looking preventive strategies is consistent with evidence from other contexts demonstrating that health and lifestyle factors require strategic planning at multiple levels. For example, Karimzadeh et al. (39)) highlight the importance of foresight-based approaches in shaping health-promoting environments, underscoring that effective health interventions must incorporate behavioral, organizational, and societal perspectives (39). Therefore, these findings provide evidence to guide national and regional health policies toward early detection, targeted health literacy interventions, and culturally responsive lifestyle modification programs to improve cerebrovascular outcomes in Thailand's aging society.

## Conclusion

This study demonstrates the interplay of biological, behavioral, and social factors in influencing stroke risk and homocysteine levels in Thai people. The statistics indicate that modifying factors such as poor dietary habits, elevated blood pressure, high bloo11d glucose, and increased homocysteine levels can significantly elevate the risk of stroke. Due to the Thai lifestyle, characterized by a high consumption of sticky rice, dependence on convenience foods, and a shift toward nuclear family models, public health interventions must be culturally sensitive, multifaceted, and comprehensive. To reduce the incidence of strokes and enhance cardiovascular health in Thailand, it is essential to address the interconnected risk factors.

## Data Availability

The datasets generated and analyzed during the current study contain sensitive personal and health-related information and are therefore not publicly available. Access to anonymized data may be considered upon reasonable request to the corresponding author, subject to approval by the institutional ethics committee.
